# The characteristics of sexual behavior in blind men in Ganzhou, China: A cross-sectional study

**DOI:** 10.1097/MD.0000000000037574

**Published:** 2024-04-26

**Authors:** Shaoming Huang, Shenghuang Zhu, Renfu Liu, Chuanchuan Xiong, Lixin Liu, Jiaguo Huang, Biao Qian

**Affiliations:** a Department of Urology, Ganzhou Hospital of Guangdong Provincial People’s Hospital, Ganzhou Municipal Hospital, Ganzhou, Jiangxi, China; b Department of Urology, The People’s Hospital of Yudu County, Ganzhou, Jiangxi, China; c Department of Urology, Affiliated Xiaoshan Hospital, Hangzhou Normal University, Hangzhou, Zhejiang, China; d Department of Urology, The First Affiliated Hospital of Gannan Medical College, Ganzhou, Jiangxi, China.

**Keywords:** blind men, questionnaire, sex education, sexual arousal, sexual behavior

## Abstract

Visual stimuli play key roles in influencing men sexual behavior. However, few studies have explored the sexual behavior of blind men. To provide more information about blind men for the study of andrology by surveying the characteristics of their current sexual behavior. A questionnaire-based cross-sectional study design was performed. The questionnaire contained questions regarding demographic characteristics of participants, access to sexual knowledge, perception of the sexual partners’ beauty, and sexual arousal. Blind men were interviewed face-to-face by the trained investigator. Complete questionnaires were collected from 54 participants, with an average age of 40.57 ± 9.80 years old. Eye diseases were the most frequent cause of blindness. In terms of sexual orientation, all participants were heterosexual. Notably, 90.7% of the participants reported to have had a sexual experience. Among those who had engaged in sexual behavior, 93.6% experienced sexual pleasure and 69.4% had a normal erectile function. Overall, 16.7% of the participants received sex education. The participants obtained sexual knowledge mainly through sounds from mobile phones, peer-to-peer communication, sounds of television and radio. Voice was the most frequent perception of the sexual partners’ beauty, followed by figure, skin, and body fragrance. In terms of stimuli of sexual arousal, tactile sensation and auditory sensation in that order were the most frequent stimuli of sexual arousal. Stimuli of sexual arousal in blind men are mainly mediated by sound and touch. Blind men understand their sexual partners’ beauty through auditory, tactile, and olfactory sensations. Blind men in Ganzhou lack formal and systematic sex education.

## 1. Introduction

Sexual arousal is divided into subjective and physiological arousal.^[1]^ Subjective arousal refers to the emotional experience that includes the awareness of self-arousal, expected feedback, and stimulated desire. Physiological arousal is driven by vasodilatation of genital tissues. Sexual arousal is influenced by neural (sensory and cognition), hormonal, and genetic factors. In humans, the culture and environment are important factor affecting sexual arousal. Generally, sexual arousal is a dynamic and highly coordinated neurophysiological process, which can be caused by visual stimuli.^[2]^ The eye is the most important sensory organ in human beings. About 80% to 90% of the information received by the human body from the external environment passes through the visual channel.^[3]^ Therefore, damage to the eyes or a complete lack of vision, impacts cognition, motivation, emotion, and autonomous responses. As with most physiological processes, sexual arousal is a multisensory experience. Although touch is the primary mediator in most sexual interactions, visual stimuli, in the form of sexual partners observing each other bodies, have also been shown to play a role in this process. Moreover, visual stimuli elicit intense sexual arousal even in the absence of tactile stimuli.^[4,5]^ Gender differences have been reported in arousal induced by visual sexual stimuli, this is based on evidence from different gaze fixation patterns and erotic images.^[6–8]^ For example, a previous study using functional magnetic resonance imaging showed that the amygdala and hypothalamus of men were more strongly activated than in women when watching the same visual sexual stimuli.^[9]^ Moreover, visual stimuli are very important for men sexual behavior. Thus, either visual impairment or complete lack of vision in men affects the subjective components of sexual response, possibly hindering physiological autonomous responses. However, few studies have explored the sexual behavior of blind men. To provide more information regarding the andrology of blind men, survey questionnaires were used to determine the stimuli that contribute to their sexual arousal, how they perceive their sexual partners’ beauty, and how they acquired sexual knowledge.

## 2. Materials and methods

### 2.1. Study design

This was a cross-sectional study with a convenience sample. Individuals diagnosed by doctors with visual impairment are registered with Chinese Disabled Persons’ Federation and the Blind Association. The Blind Association is a nonprofit social organization founded by national blind and social organizations, enterprises, institutions, and individuals working for the blind. Local blind men in Ganzhou, directors of the Disabled Persons’ Federation and the Blind Association of Ganzhou were contacted and asked to participate in the study. Prior to completing the questionnaires, blind men were briefed on the voluntary and anonymous nature of their participation, with the assurance that they could withdraw from the study at any point. The study was approved by the Ethical Committee of Ganzhou Municipal Hospital.

### 2.2. Participant recruitment

Recruitment for the surveys took place through the roster provided by the Disabled Persons’ Federation and the Blind Association of Ganzhou. Blind men who meet the inclusion and exclusion criteria were first telephoned by the local chapter of the Disabled Persons’ Federation. Inclusion criteria were as follows: individuals with grade 1 and grade 2 visual disabilities, all of whom were men over 18 years old and participated voluntarily. The following exclusion criteria were applied: Individuals who also had severe organic diseases (cerebral infarction, liver failure, chronic renal failure, deaf or mute, deaf or mute, etc); Individuals with obvious genital deformities (anorchism, genital defects, micropenis that unable to complete sexual intercourse, etc); Homosexuality or bisexuality.

In China, visual disability is diagnosed and identified either by a government-designated disability institution or a tertiary hospital. Visual disability is divided into 4 levels according to the international classification. Individuals who have a best-corrected visual acuity of < 0.02 or visual field radius < 5° in the better-seeing eye are classified as grade 1, those with best-corrected visual acuity of 0.02~<0.05 or visual field radius < 10° in the better-seeing eye as grade 2, those with best-corrected visual acuity of 0.05~<0.1 in the better-seeing eye as grade 3, those with best-corrected visual acuity of 0.1~<0.3 in the better-seeing eye as grade 4. If eyes have different vision, the one with better vision is selected. The best-corrected vision refers to the best vision that can be achieved by correcting with appropriate lenses, or the vision measured by a pinhole mirror. Those with a visual field radius of <10 ° are considered blind regardless of their visual acuity.^[10,11]^

### 2.3. Survey questionnaire design

The survey questionnaire was designed as per principles of purpose, acceptability, order, conciseness, and matching. It was divided into preliminary and formal surveys.

### 2.4. Preliminary survey

In the preliminary survey, researchers first went to the potential blind participants to seek informed consent and voluntary participation. Then, through face-to-face Question and Answer sessions, the researchers obtained general demographic information including name, age, sexual orientation, marital status, causes of blindness and specific sexual life conditions such as stimuli of sexual arousal, the perception of sexual partner beauty, and the acquisition of sexual knowledge using open questions. For each participant, erectile function was evaluated in the past 4 weeks according to the international erectile function score (IIEF-5 score).

### 2.5. Formal questionnaire

Through the preliminary survey and the discussion of the team, a formal questionnaire was generated and reviewed by the Ethics Committee and was deemed reliable and valid by experts in the field. The divided into 2 sections: demographic characteristics of participants, sexual behavior characteristics of participants. Some of the survey questions were single-answer questions, some were multiple-answer questions and others questions were subjective.

The first part of the questionnaire contained questions related to demographic characteristics of participants such as name, age, sexual orientation and marital status as well as the cause of your blindness.

The personal sexual behavior characteristics section of the questionnaire contained questions meant to collect information about sexual arousal stimuli, the perception of beauty of their sexual partner, access to sexual knowledge, sex education experience, age of first sexual experience, frequency of sexual activity, experiences in sexual pleasure and evaluation of erectile function:

- What is the source of your sexual arousal?
- What is your perception of the beauty of your sexual partner from many aspects?
- How do you get sexual knowledge?
- Have you ever received formal and systematic sex education?
- What is your age of first sexual behavior?
- What is your sexual frequency?
- Have you ever had sexual pleasure?
- Please evaluate your erectile function in the past 4 weeks, according to the IIEF-5 score.

### 2.6. Investigation procedure

First, written informed consent was obtained from all participants and their legal guardians. Some blind men could sign their own names. If they were unable to sign it personally, their legal guardians were asked to sign. All personnel participating in this study had undergone prior training on unifying inquiry standards and inspection methods and clarifying precautions in the investigation. Blind men were organized to collectively meet at the hospital or designated places, where individual face-to-face Question and Answer sessions based on the survey questionnaire were conducted. Questionnaire and IIEF-5 score were read by the investigator to the blind men and scored according to the answers.

### 2.7. Data collection and analysis

The data obtained from questionnaires were reviewed by the members of the research group and entered into Excel worksheets, which were then used to create a database in EpiData 3.1 software (Odense, Denmark). Descriptive analysis was performed on the responses. Descriptive data are presented as means ± standard deviations, and categorical variables are presented as frequencies and percentages.

## 3. Results

### 3.1. Demographic characteristics of participants

According to age, visual disability grade and exclusion criteria, we sent a questionnaire to 67 blind men who met the criteria. A total of 54 participants returned a completed survey whose ages ranged from 19 to 62 years of age, with an average age of 40.57 ± 9.80 years old. Their sexual orientation was all heterosexual. Those who were married were 66.7%, unmarried were 29.6%, and divorced or widowed were 3.7%. Eye diseases (50.0%) were the most frequent causes of blindness, followed by congenital blindness (18.6%), trauma (14.8%), high fever (11.0%), and unexplained blindness (5.6%) (Table 1).

**Table 1:** Demographic characteristics of participants.

	Number (n)	Percentage (%)
Marital status		
Married	36	66.7
Unmarried	16	29.6
Divorced or widowed	2	3.7
Causes of blindness		
Eye disease	27	50.0
Retinopathy	16	29.6
Maculopathy	7	13.0
Glaucoma	2	3.7
Optic neuropathy	2	3.7
Congenital blindness	10	18.5
Trauma	8	14.8
High fever	6	11.1
Unexplained blindness	3	5.6

### 3.2. Sexual behavior characteristics of participants

Among the 54 blind men, virgins accounted for 9.3% of the participants. In terms of sexual frequency, 25.9%, 11.1%, 11.1%, and 51.9% of blind men had engaged in sexual behavior 1 to 4, 4 to 8, >8, and <1 times a month, respectively. For 16.7%, 57.4%, 11.0%, and 5.6% of the 49 participants, the age of first sexual behavior — sexual behavior is defined as vaginal intercourse — were younger than 20 years old, 21 to 25 years old, 26 to 30 years old, and older than 30 years old, respectively.

Among those who reported to have engaged in sexual behavior (n = 49), 93.6% experienced sexual pleasure (orgasms) whereas 6.4% did not. 69.4%, 16.3%, 10.2%, and 4.1% had normal erectile function (IIEF-5 score ≥ 22), mild (IIEF-5 score 12–21), moderate (IIEF-5 score 8–11), and severe erectile dysfunction (IIEF-5 score < 7), respectively. The most authoritative international questionnaire for evaluation of erectile dysfunction is the International Index of erectile function (IIEF), which was originally designed by Rosen in 1997 and had 15 questions but was later simplified into 5 questions (IIEF-5). Therefore, survey questions on erectile dysfunction were based on the IIEF-5 score as per sexual activity in the past 4 weeks. The scores are classified into normal erectile function (IIEF-5 score ≥ 22), mild erectile dysfunction (IIEF-5 score = 12–21), moderate erectile dysfunction (IIEF-5 score = 8–11) and severe erectile dysfunction (IIEF-5 score < 7).

Participants accessed sexual knowledge through sounds from mobile phones (self-media) (51.9%), peer-to-peer communication (48.1%), sounds from television (35.2%), and radio (27.8%). What was most frequently defined as beauty in a sexual partner was voice (64.8%), figure (55.5%), skin (51.9%) and body fragrance (18.5%). The most arousing sexual stimuli were: tactile sensation (53.7%), auditory sensation (40.7%) and mood (22.2%) (Table 2).

**Table 2:** Sexual behavior characteristics of participants.

	Number (n)	Percentage (%)
Age of first sexual behavior		
≤20 yr old	9	16.7
21–25 yr old	31	57.4
26–30 yr old	6	11.1
>30 yr old	3	5.6
None	5	9.2
Sexual frequency		
>8 times a mo	6	11.1
4–8 times a mo	6	11.1
1–4 times a mo	14	25.9
Less or none	28	51.9
Sexual pleasure		
Sexual pleasure	47	87.1
Asexual pleasure	2	3.7
Asexual behavior experience	5	9.2
Erectile function		
Normal	34	69.4
Mild erectile dysfunction	8	16.3
Moderate erectile dysfunction	5	10.2
Severe erectile dysfunction	2	4.1
Receive sex education	9	16.7
Access to sexual knowledge		
Sounds from mobile phones (self-media)	28	51.9
Peer-to-peer communication	26	48.1
Sounds from television	19	35.2
Radio	15	27.8
Perception of beauty of sexual partner		
Voice	35	64.8
Figure	30	55.5
Skin	28	51.9
Body fragrance	10	18.5
Sexual arousal		
Tactile sensation	29	53.7
Auditory sensation	22	40.7
Mood	12	22.2

## 4. Discussion

In our research, we discovered that blind men primarily acquire sexual knowledge through auditory cues from mobile phones, interpersonal communication, and audio content from television and radio. The factors that induce sexual arousal in blind men predominantly revolve around auditory and tactile stimuli. Regarding their preferences in sexual partners, blind men are influenced by qualities that can be heard, touched, and smelled. Notably, blind men often face a deficiency in formal and systematic sex education. To date, no study has explored the sexual behavior of blind people. This is likely, due to the small proportion of blind people in the population. Given that China is a populous country, such social problems cannot be ignored. Due to their unique physiological and psychological characteristics that differ from normal people, individuals with disabilities may experience complex sexual challenges in various aspects such as ethics, morality, physiology, psychology, and society.^[12–14]^ For example, the hearing impaired seldomly encounter a full sexual experience due to hearing and speech communication barriers.^[15]^ Thus, the higher their disability level the lower their sexual satisfaction.^[15]^ Blindness or acquired blindness, the lack of sensory impulses from the eyes affects sexual activities.^[16,17]^ Although numerous studies have explored sexual behaviors in individuals with unimpaired vision, research on sexual behavior among the blind is largely lacking.^[2,18,19]^ Thus, to provide more information about blind men for the study of andrology, we surveyed blind men to reveal characteristics of sexual behavior in blind men.

In previous studies, sexual arousal in the general population is an emotional state produced through the processing of both external sexual stimuli such as visual and auditory sexual stimuli, sexual caresses, and internal sexual stimuli such as sexual fantasies.^[20]^ In sexual cognition, the visual sense is the first important channel through which people receive sexual information^[17]^ as human visual perception develops fastest and most sufficiently among the senses. Investigating whether there are differences in sexual psychology and cognition between blind men, particularly those with congenital blindness, and the general population will provide important scientific data for this population. Consequently, we conducted a questionnaire survey to identify the factors that predominantly influence sexual arousal in blind men, revealing that hearing and touching play a crucial role in determining these stimuli. This corroborated the definition of sexual arousal as an emotional state created by an individual by processing either external, such as visual and tactile stimuli, or internal such as fantasies, sexual stimuli.^[17,21,22]^ Thus, the blind adapt to the use of other senses, – hearing, touch, taste, smell, and proprioception – to better obtain information from the surrounding environment, causing these senses to become unusually sharp.^[17]^

Blind men define beauty in their sexual partners through hearing, touch, and smell corroborating the hypothesis of Wu Jianhui and colleagues that those blind due to visual impairment gradually form their characteristic behavioral abilities in their interaction with the surrounding environment.^[23]^ In addition to the high sensitivities in hearing and touch, the plasticity of the central nervous system undergoes changes to accommodate the distinctive sensory world of the blind. Our findings are consistent with reported by Song Yiqi and colleagues. In their study, they demonstrated that the limited utilization of the visual channel in blind students prompts the development of specialized compensatory mechanisms through tactile channel, supporting the principles of perceptual symbol theory.^[24]^ However, we were surprised that smell was least used in the perception of beauty, despite its importance in promoting sexual and mating behavior and even romantic relationships. This may be related to the promotion process of sexual behavior whose process often starts with suggestive words and contact actions, with the sense of taste only coming in, after further intimate actions.

Blind men had their first sexual encounter at an age later than that of the general population and had a lower frequency of sexual behavior than the general population. The average age at first sexual encounter among Chinese young people aged 20 to 29 is 21.88 years,^[25]^ whereas, for blind men aged 20 to 29 in our study, it was 22.98 years – this difference is possibly closely related to the social environment and poor systematic sex education. A survey conducted by Chinese scholars and supported by the United Nations Educational, Scientific and Cultural Organization showed that disabled adolescents faced many obstacles in accessing sex education and reproductive health services, especially in rural areas.^[26]^ Two other studies on the status of sex education for disabled students in special education schools in Xinjiang, China, also showed that sex education was lagging behind and was difficult to carry out.^[27,28]^

In this study, similar to the general population, we found that blind men often achieved orgasm. On the other hand, they had different degrees of erectile dysfunction. As a cross-sectional study based on a questionnaire, we were only able to assess the erectile function of the participants by the IIEF-5 score. Due to the complex etiology of erectile dysfunction and the small sample size, we did not analyze the causes of erectile dysfunction in this study. Although we can infer the cause of erectile dysfunction through the cause of blindness or the patient basic diseases. For example, retinopathy caused by diabetes mellitus may lead to blindness, and diabetes is also a risk factor for erectile dysfunction. However, we infer that such an inference may be weak. However, many blind men with erectile dysfunction did not seek formal diagnosis and further medical treatment, mainly due to the inconvenience of medical treatment; a considerable number of people felt that this was not a disease and thus did not need medical treatment. This perception is possibly attributed to poor systematic sex education. Wan Xing and colleagues reported that disabled adolescents face a more severe sexual and reproductive health situation than ordinary adolescents, and their disability makes it difficult for them to obtain equal educational opportunities, lack the ability of self-protection, and be vulnerable to sexual and reproductive health problems.^[29]^ Hence, this research reveals important information that increases awareness and education regarding appropriate sexual behavior, highlights the significance of understanding sexual conduct among individuals with visual impairments, as well as relationship between sexual physiology and mental health. Generally, men have a stronger response to visual sexual stimuli than women, and men are thus more exposed to all kinds of pornographic networks, magazines, newspapers, and periodicals than women.^[17]^ In today open and tolerant culture, the sexual behavior of people in various societal groups affects not only the happiness of individuals and families but also the stability of the whole society. This survey found that the blind men access to sexual knowledge mainly comes from sounds from their mobile phones radio and television, and peer-to-peer communication.

Due to the limited sample size and survey content of this study, only preliminary analyses of visual, tactile, and auditory aspects of sexual arousal were built upon in subsequent steps. The comparison of men who were blind due either to congenital blindness or acquired blindness with the general population is a worthwhile study to conduct. Furthermore, it is imperative to conduct a comparative analysis between blind men and other individuals with disabilities, such as those who are deaf or but have normal vision. Exploring potential distinctions between men with congenital blindness and those who acquired blindness after puberty is also advocated. Additionally, the underlying causes of erectile dysfunction among blind men is poorly understood. A more detailed analysis is necessary to understand the factors contributing to erectile dysfunction and discuss their potential impact on participants, potential interventions or implications for clinical practice. However, we could not conduct grouping analysis due to the limited number of respondents in this survey. There are still some limitations in our conclusions, such as the lack of formal and systematic sex education in the study population, their cultural diffrences among others. These will be considered in our future investigations.

## 5. Conclusion

The stimuli of sexual arousal in blind men are mainly mediated through sound and touch. Blind men define their sexual partner beauty through hearing, touch, and smell. Finally, visually impaired individuals in Ganzhou lack access to formal and systematic sex education. Ganzhou, being a relatively developed city, shares similarities with numerous other cities across China. Therefore, our research sheds light on the cultural norms prevalent among blind men in such urban settings. Undeniably, this study offers valuable insights for understanding andrology diseases among the blind male population.

## Acknowledgments

We acknowledge the contribution of the participants. We thank Home for Researchers editorial team (www.home-for-researchers.com) for language editing service.

## Author contributions

**Conceptualization:** Biao Qian.

**Data curation:** Shenghuang Zhu, Renfu Liu, Lixin Liu.

**Formal analysis:** Chuanchuan Xiong, Lixin Liu.

**Funding acquisition:** Shaoming Huang.

**Investigation:** Shenghuang Zhu, Renfu Liu.

**Software:** Jiaguo Huang.

**Supervision:** Biao Qian.

**Writing – original draft:** Shaoming Huang.

**Writing – review & editing:** Jiaguo Huang, Biao Qian.
